# Mindfulness Training Enhances Endurance Performance and Executive Functions in Athletes: An Event-Related Potential Study

**DOI:** 10.1155/2020/8213710

**Published:** 2020-08-28

**Authors:** Jui-Ti Nien, Chih-Han Wu, Kao-Teng Yang, Yu-Min Cho, Chien-Heng Chu, Yu-Kai Chang, Chenglin Zhou

**Affiliations:** ^1^Graduate Institute of Athletics and Coaching Science, National Taiwan Sport University, Taoyuan City, Taiwan; ^2^Center for East-West Medicine, David Geffen School of Medicine, University of California, Los Angeles, California, USA; ^3^Center for Educational Resources, Buddhist Tzu Chi Medical Foundation, Los Angeles, California, USA; ^4^Department of Physical Education, National Taiwan Normal University, Taipei, Taiwan; ^5^Institute for Research Excellence in Learning Science, National Taiwan Normal University, Taipei, Taiwan; ^6^School of Kinesiology, Shanghai University of Sport, Shanghai, China

## Abstract

Mindfulness interventions have been linked to improved sport performance and executive functions; however, few studies have explored the effects of mindfulness on sport performance and executive functions simultaneously. This study sought to examine whether a mindfulness training program would affect both the endurance performance and executive functions of athletes. In addition, event-related potentials (ERPs) associated with the Stroop task were assessed to investigate the potential electrophysiological activation associated with the mindfulness training. Applying a quasiexperimental design, forty-six university athletes were recruited and assigned into a five-week mindfulness training program or a waiting list control group. For each participant, the mindfulness level, endurance performance assessed by a graded exercise test, executive functions assessed via Stroop task, and N2 component of ERPs were measured prior to and following the 5-week intervention. After adjusting for the preintervention scores as a covariate, it was found that the postintervention mindfulness level, exhaustion time, and Stroop task accuracy scores, regardless of task condition, of the mindfulness group were higher than those of the control group. The mindfulness group also exhibited a smaller N2 amplitude than the control group. These results suggest that the five-week mindfulness program can enhance the mindfulness level, endurance performance, and multiple cognitive functions, including executive functions, of university athletes. Mindfulness training may also reduce conflict monitoring in neural processes.

## 1. Introduction

Mindfulness, a concept that originated from Buddhist meditation practices and was later developed and adapted by various scholars [1, 2], has been described as “the awareness that emerges through paying attention on purpose, in the present moment, and nonjudgmentally to the unfolding of experience moment by moment” ([3], p. 145) and “a process of openly attending, with awareness, to one's present moment experience” ([4], p. 493), with the essential processes of mindfulness involving awareness, open-minded attention, being present in the moment, and nonjudgment. Mindfulness can be cultivated through thought training, and accumulated rigorous evidence has indicated that mindfulness-based interventions can enhance physical health, mental health, cognitive and affective outcomes, and interpersonal outcomes [4].

Successful sport performances in high-level competitions require harmony among the given athlete's physiological, psychological, and interpersonal capacities and readiness, with mindfulness potentially affecting an athlete's ability to achieve peak performances. Indeed, mindfulness-associated benefits have not only been observed in clinical and nonclinical populations [4–6] but also in specific populations dedicated to the pursuit of excellence in a given field, such as athletes [7, 8]. Specifically, mindfulness has been found to be positively associated with flow in athletes, regardless of gender or sport type [9, 10], and mindfulness-based interventions have been demonstrated to enhance sport-associated physiological activations (e.g., salivary cortisol levels and immune responses) and psychological status indicators (e.g., flow and anxiety reduction), as well as sport performances themselves (e.g., shooting and dart throwing performances) [7]. It should be noted, however, that the majority of sport studies regarding mindfulness have emphasized either mindfulness-related physiological or psychological aspects of fine motor performance (e.g., shooting), with few studies investigating gross motor performance (e.g., running) [11].

Executive functions can be described as the higher order or metacognitive functions that are utilized to regulate a self-directed set of purpose-oriented actions in novel or nonhabitual situations [12, 13]. These functions, which also enable an individual to flexibly regulate and control his or her own mind and behavior to achieve goals through the operation of fundamental cognitive processes [14], may be regarded as a significant characteristic of elite athletes. For example, athletes, regardless of whether they play self-paced sports (e.g., golf, bowling, and archery) or externally paced sports (e.g., tennis, baseball), have been found to achieve higher scores on executive function-related tasks than nonathletes [15]. Better executive functions have also been found to distinguish talented from amateur players with 89% accuracy [16] and to differentiate elite and subelite youth players, even after adjusting for training hours [17], reflecting the likelihood that executive functions are a crucial indicator for athletic success.

Mindfulness-based interventions have been linked to increased executive functions [18]. Moynihan et al. [19] observed a lower Trails B/A ratio for the Trail Making Test, a positive executive functions index, in older adults following mindfulness-based stress reduction (MBSR) relative to waiting list controls. Additionally, several studies involving the Stroop task, the neuropsychological test most commonly used to assess executive functioning, have also shown that executive functions are link to mindfulness training. For example, Teper and Inzlicht [20] found that more meditation practice is associated with greater executive functioning, as reflected by fewer errors on the Stroop task among meditators than among nonmeditators, while Allen et al. [21] observed improvements in Stroop conflict among adults who attended a six-week mindfulness training program as compared to a control group. The improved executive functions resulting from mindfulness may be mediated by mindfulness-induced brain plasticity. For example, mindfulness training produces larger no-go N2 event-related potential (ERP) amplitudes, an electrophysiological index of ERPs, which indicates better inhibitory control of behavioral goal-prepotent response tendencies [22]. Similarly, Teper and Inzlicht [20] observed a mediating role of the error-related negativity (ERN) amplitude of ERPs between mindfulness experience and Stroop performance. Given that the ERN amplitude has been linked to the anterior cingulate cortex, a brain region implicated in executive functions, these findings suggest that mindfulness affects specific brain regions to enhance executive functioning. The aforementioned studies demonstrated that mindfulness is associated with executive functions, and investigations of ERPs have further indicated the underlying mechanisms of brain modifications from an electrophysiological perspective. However, whether mindfulness is associated with executive functions in athletes remains unknown because no studies to date have examined the relationship between mindfulness and executive functions or potential underlying mechanisms in athletic populations.

Therefore, the purpose of the present study was to explore the effects of mindfulness-based training on sport performance via endurance/gross motor performance (i.e., maximum oxygen consumption (VO_2max_) and exhaustion duration) in trained athletes. Additionally, the effects of the mindfulness training were also examined with respect to executive functions from both behavioral and electrophysiological perspectives, with an emphasis on the N2 component of ERPs during the Stroop task. It was hypothesized that a group of participants who took part in mindfulness training would exhibit better endurance performance after that training than a control group, as well as better executive function performance and larger N2 amplitudes.

## 2. Methods

### 2.1. Participants

Forty-six athletes aged 18-25 years old were recruited from two of National Taiwan Sport University's sports teams, Taiwan. Potential participants were screened and only included if they met the following criteria: (a) no prior experience in mindfulness meditation or other types of meditation, (b) normal or corrected-to-normal 20/20 vision, (c) no red-green color blindness, (d) no history of neurological disorders or cardiovascular disease, and (e) passing the criteria of the physical activity readiness questionnaire (PAR-Q) to ensure the ability to safely perform the cardiorespiratory fitness exercise test. Each participant's verbal short-term memory was assessed by the Digit Span test of the Wechsler Adult Intelligence Scale-Third Edition [23]. The participants were assigned into either the mindfulness training group (*n* = 23; 16 males, 7 females) or the waiting list control group (*n* = 23; 18 males, 5 females) based upon their voluntary willingness.  Table 1 summarizes the participants' demographic information. This study, in accordance with the requirements of the Declaration of Helsinki, was approved by the Institutional Review Board of Fu Jen Catholic University.

### 2.2. Measures

#### 2.2.1. Graded Exercise Test (GXT)

Each participant's endurance performance (i.e., VO_2max_ and exhaustion time) was assessed via the GXT using a motor-driven treadmill (pulsar, h/p/cosmos, Germany). The GXT has been widely used to examine the dynamic relationships between exercise and integrated physiological systems [24]. During the GXT, the given participant's VO_2_ and respiratory exchange ratio were recorded by a computerized indirect calorimetry system (SensorMedics Vmax 29C, USA). The initial treadmill speed of the GXT was 2.0 m/s with increases of 0.5 m/s every 2 min until the participant reached volitional exhaustion. During the last 20 seconds of each 2 min stage, the heart rate and rating of perceived exertion (RPE) were recorded using a Polar heart rate monitor (Sport Tester PE 3000, Polar Electro Oy, Kempele, Finland) and the Borg 6 to 20 scale [25], respectively. The VO_2max_ was determined at the point of volitional exhaustion as evidenced by meeting two of the following three criteria: (1) the RPE score was >18, (2) the HR was ≥90% of the age-predicted maximum heart rate (HR_max_), and (3) the respiratory exchange ratio was >1.10 [26, 27]. The values of the VO_2max_ and time-to-exhaustion were recorded as the main index for the sport performance.

#### 2.2.2. Stroop Task

The modified computerized Stroop task [28], a widely utilized neuropsychological test of both basic information processing and executive functions [29, 30], was conducted using Neuroscan STIM2 software (Neurosoft Labs Inc., Sterling, VA, USA).

The Stroop task consists of three types of trials (i.e., neutral, congruent, and incongruent trials). In the version of the task used in this study, the neutral trials displayed squares of one of three colors (i.e., red, green, or blue), and the congruent trials displayed one of three Chinese color words (i.e., 藍 (blue), 綠 (green), or 紅 (red)) in pixels of the same color (e.g., the word 藍 (blue) in blue pixels). Finally, the incongruent trials also displayed the three Chinese color words, but the color of the pixels in which each word was shown were inconsistent with the semantic meaning of the word (e.g., the word 藍 (blue) was presented in red pixels). Each type of trial and the various colors of the stimuli were presented randomly with identical frequency (e.g., there were 36 congruent trials and one-third of these were displayed in red pixels).

During the task, each stimulus was presented at the center of a 17-inch computer screen for 500 ms, which was then followed by an intertrial interval of 2000 ms. For each stimulus, only a correct response made between 200 ms and 1000 ms after the initial stimulus presentation was considered for further analysis. Responses made outside this 200-1000 ms time window, incorrect responses, or a lack of response were all considered incorrect responses.

Each participant was instructed to respond according to the actual color of the presented stimulus itself by pressing one of the three color response buttons on a (10 cm × 8 cm × 2 cm) response box as quickly and accurately as possible. Five blocks with each block consisting of 108 trials were conducted during the experiment. The total length of the Stroop task in this study was approximately 30 min.

#### 2.2.3. Mindfulness Level

The Chinese version of the Mindful Attention Awareness Scale (CMAAS) [31], which is based on the Mindful Attention Awareness Scale (MAAS) [32], was utilized to assess individual differences in dispositional mindfulness over time. The CMAAS is a 15-item questionnaire that includes items such as “It seems I am “running on automatic” without much awareness of what I'm doing” and “I find myself doing things without paying attention.” All the items responded to using a 6-point Likert scale ranging from 1 (“almost always”) to 6 (“almost never”). Items on the CMAAS are reverse scored such that higher scores indicate high dispositional mindfulness. The CMAAS has been shown to have high internal consistency and test-retest reliability [31], with Cronbach's *α* values between pre- and posttests (e.g., tests conducted with a 5-week interval in between) ranging between 0.83 and 0.85.

### 2.3. Mindfulness Training Program

The mindfulness training program used in this study was developed based on the original mindfulness program [33] and mindful sport performance enhancement (MSPE) [11, 34]. In addition, stress coping techniques and techniques relating to the application of mindfulness in a sports competition context were practiced during group discussions in the mindfulness training program.

Given past suggestions regarding effective mindfulness training [7], the mindfulness program consisted of two 30 min sessions per week for 5 weeks. The participants were also strongly encouraged to practice for at least 15-30 min per day between the sessions. During the first 5 sessions, the concepts (i.e., focusing attention on experiences with nonjudgment in the present moment) and components (i.e., awareness and acceptance) of mindfulness were introduced. The relative mindfulness skills, including mindful breathing, mindful meditation, body scanning, mindful yoga, and mindful walking [3], were also introduced during the first 5 sessions. In addition to practicing the above mindfulness skills, the application of mindfulness during sports competition situations were subsequently introduced as of the 6^th^ session. The program was conducted in a training center of National Taiwan Sport University, Taiwan. Appendix presents for a summary outline of the five-week mindfulness program protocol.

### 2.4. Event-Related Potential Recording and Analysis

The continuous electroencephalogram (EEG) activity of each participant was recorded throughout the entire Stroop task using a Neuroscan Quick-Cap with 32 Ag/AgCl electrodes placed according to the International 10/20 system (Neuroscan Quick-Cap, Neuroscan Inc., VA). The montage was referenced offline to the average right and left mastoid, and the FPz electrode served as the ground. The interelectrode impedance was maintained below 10 k*Ω*. The activities of horizontal and vertical electrooculogram (EOG) were recorded by the two electrodes placed above and below the left eye orbits and at the outer canthus of each eye. The EEG activity was amplified using a Neuroscan Synamps2 amplifier (Scan 4.5, Neurosoft Labs, Inc.) with a sampling rate of 500 Hz. Additionally, a notch filter at 60 Hz was applied to eliminate any potential artifacts. During the entire Stroop task, each of the participants was comfortably seated in a chair in a sound-proof room.

The offline EEG activity from the correct response trials was then segmented into epochs from 100 ms prestimulus onset to 1000 ms poststimulus onset. Baseline correction was conducted using the 100 to 0 ms prestimulus interval and then filtered using a zero-shift low-pass cutoff at 30 Hz (12 dB/oct). Based on a visual inspection of the averaged grand waveform, the time windows for each ERP component were determined. The N2 amplitude was calculated by averaging the mean amplitudes of the F3, Fz, and F4 electrodes as frontal region during the time window from 235-345 ms after the stimulus onset.

### 2.5. Procedure

On the first visit, each participant or their legal guardians completed a written informed consent form. On first arriving at the laboratory, each participant was escorted into a sound-proof room for the recording of his or her EEG activity as the Stroop task was conducted. Then, the mindfulness level and sport performance of each participant were assessed using the CMAAS and the GXT, respectively. During the 5-week intervention period, the participants in the mindfulness group were prompted to attend the sessions of the mindfulness program (twice per week for five weeks) before their daily sports training in a training center, and the athletes in the control group maintained their daily routines. The sports performance and Stroop task performance of all the participants, as well as their mindfulness levels, were then assessed at the postintervention time point (i.e., at the end of week 5). Each participant was compensated with approximately US $50 for participating in the experiment.

### 2.6. Statistical Analysis

Descriptive statistics (performed using SPSS version 20, IBM Corp., Armonk, NY, USA) were utilized to calculate the means and standard deviations of the demographic data and outcomes. The postintervention mindfulness level scores of the mindfulness and control groups were compared using a one-way analysis of covariance (ANCOVA), with the preintervention scores as the covariate. Separate ANCOVAs, adjusted for the preintervention scores, were also employed to compare the post-intervention endurance performance (i.e., VO_2max_ and exhaustion time) and executive function scores (i.e., accuracy and response time for the Stroop task), as well as the ERP results (i.e., the averaged N2 mean amplitude of Fz, F3, and F4), of the two groups to determine whether the mindfulness intervention resulted in any differences between the groups. It is suggested that the ANCOVA be utilized in experimental designs to avoid bias from treatment effects [35–37]. The effect size via partial *η*2 was calculated for the variability between groups when a significant effect was found. A *p* value less than 0.05 was considered statistically significant.

## 3. Results

### 3.1. Participant Characteristics

Independent sample *t*-testing revealed no significant between-group differences in the majority of variables, except for height (*p* < 0.05), as of the preintervention testing (*p* > 0.05) (see Table 1).

### 3.2. Measures

Table 2 presents the raw descriptive statistics of the outcome measures from the pretest to the posttest for the mindfulness and control groups in terms of the dispositional mindfulness, GXT (VO_2max_ and exhaustion duration), Stroop task (accuracy and response time), and N2 amplitude.

#### 3.2.1. Mindfulness Level

**Figure 1:**
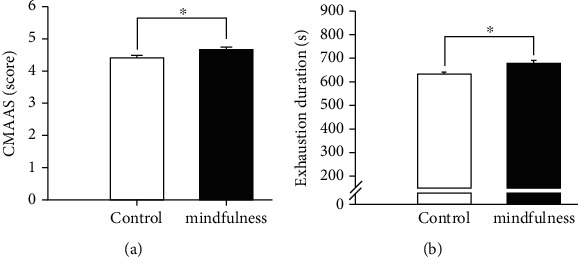
Postintervention scores (*M* ± SE) between the mindfulness and control group after controlling for the preintervention scores for the (a) Chinese version of the Mindful Attention Awareness Scale (CMAAS) of mindfulness level and (b) exhaustion duration of endurance performance.

The one-way ANCOVA results showed a significant postintervention CMAAS score difference between the mindfulness and control groups, *F* (1, 43) = 5.16, *p* = 0.03, and partial *η*2 = .11, with the mindfulness group having a greater CMAAS score (*M* = 4.69, SE = 0.09) than the control group (*M* = 4.40, SE = 0.09) after controlling for the preintervention CMAAS scores (Figure 1(a)).

#### 3.2.2. Endurance Performance

The one-way ANCOVA results showed no significant posttest VO_2max_ differences between the mindfulness and control groups, *F* (1, 43) = 0.08, *p* = 0.78, were observed. However, the one-way ANCOVA results in a significant postintervention exhaustion duration difference between the mindfulness and control groups, *F* (1, 43) = 15.39, *p* = 0.001, and partial *η*2 = 0.26, with the mindfulness group (*M* = 682.62, SE = 8.50) having a longer exhaustion duration than the control group (*M* = 632.95, SE = 8.50) after controlling for the preintervention exhaustion duration (Figure 1(b)).

#### 3.2.3. Executive Functions

**Figure 2:**
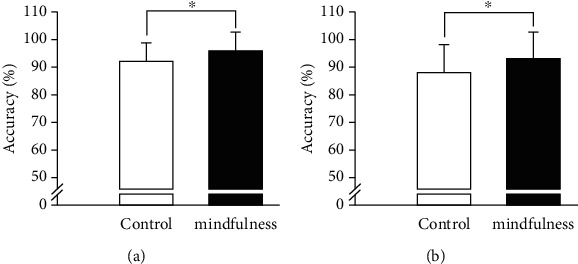
Postintervention scores (*M* ± SE) between the mindfulness and control group after controlling for the preintervention scores for the (a) Stroop congruent condition and (b) Stroop incongruent condition.

Regarding the congruent condition of the Stroop task, the one-way ANCOVA results showed a significant postintervention accuracy difference between the mindfulness and control groups, *F* (1, 43) = 11.04, *p* = 0.002, partial *η*2 = 0.20, with the mindfulness group (*M* = 0.96, SE = 0.07) having greater accuracy than the control group (*M* = 0.92, SE = 0.07) after controlling for the preintervention accuracy results. However, no significant difference between the groups was observed for the postintervention response time, *F* (1, 43) = 1.13, *p* = 0.29 (Figure 2(a)).

Regarding the incongruent condition of the Stroop task, the one-way ANCOVA results showed a significant postintervention accuracy difference between the mindfulness and control groups, *F* (1, 43) = 13.05, *p* = 0.001, and partial *η*2 = 0.23, with the mindfulness group (*M* = 0.93, SE = 0.10) having greater accuracy than the control group (*M* = 0.88, SE = 0.10) after controlling for the preintervention accuracy results. However, no significant difference between the groups was observed for the post-intervention response time, *F* (1, 43) = 2.90, *p* = 0.10 (Figure 2(b)).

#### 3.2.4. N2 Amplitude

**Figure 3:**
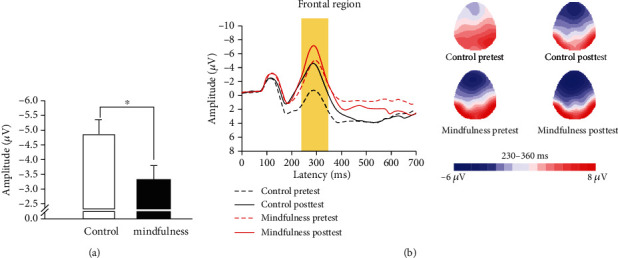
(a) Postintervention scores (*M* ± SE) between the mindfulness and control group after controlling for the preintervention scores for N2 amplitude of event-related potential. (b) Grand average of event-related potential at the frontal region across congruency in the group and time point as well as topographic scalp distribution of the N2 amplitude collapsed across congruency in group and time point.

Regarding the congruent condition of the Stroop task, no significant difference in postintervention N2 amplitudes between the groups was observed, *F* (1, 43) = 1.87, *p* = 0.18. Regarding the incongruent condition, the one-way ANCOVA results showed a significant posttest N2 amplitude difference between mindfulness and control groups, *F* (1, 43) = 4.42, *p* = 0.04, partial *η*2 = 0.09, with the mindfulness group (*M* = −3.32, SE = 0.50) exhibiting a smaller N2 amplitude than the control group (*M* = −4.87, SE = 0.50) (Figure 3).

## 4. Discussion

The current study is among the first to examine how mindfulness training simultaneously affects gross sport performance as well as tasks associated with executive functions in trained athletes from both behavioral and electrophysiological perspectives. The primary results revealed that the mindfulness training group had significantly higher mindfulness levels and exhaustion durations of endurance performance at the postintervention time point than the control group. In addition, for both the congruent and incongruent conditions of the Stroop task, the mindfulness group demonstrated higher accuracy levels but not better response times at the postintervention time point, as well as lower N2 amplitudes in the incongruent condition, than the control group.

### 4.1. Mindfulness Level

Our finding of increased mindfulness levels following mindfulness training is consistent with previous studies involving populations with cancer [38], breast cancer [39], psychosis [40], and fibromyalgia [41], as well as athletic status [7]. With regard to sports, higher mindfulness levels were observed in previous studies in university athletes following a 6-week program [42] and in Wushu athletes following an 8-week program [43]. Along with these studies, our study not only indicates that athletes can increase their mindfulness by practicing specific forms of mindfulness training but also demonstrates the effectiveness of the particular 5-week mindfulness program we proposed. Our mindfulness program may, with its relatively short length (i.e., 5 weeks on two days per week), attract athletes who might not be attracted to longer programs due to their demanding training schedules. Meanwhile, enhanced mindfulness is also encouraged for athletes because mindfulness levels have also been positively linked to stress reduction via lower salivary cortisol levels [44], the elicitation of external strategies related to better movement control, and sports-associated flow status [9, 45, 46].

### 4.2. Endurance Performance

One strength of the present study was that it investigated actual sport performance from a gross motor perspective. The observed enhanced sport performance of the mindfulness group following mindfulness training compared to the control group was similar to the findings of previous studies focusing on fine motor performance (e.g., basketball free throw shooting [47]), dart throwing [48], and shooting performance [42, 44]. While some studies have focused on running-related performance (e.g., 800-meter personal best times [49] and long-distance runners' best mile times [50]), our study advances the current knowledge regarding study design, sample size, and precise measures in the laboratory. That is, rather than applying a cross-sectional design [49] or perform a follow-up study [50], we employed a quasiexperimental design to examine the performance change resulting from mindfulness training by comparing two groups. Additionally, runners in an MSPE program in a previous study were found to show no improvements in actual running performance compared to a waiting list group, but that result may have been caused by the small sample size in that study (i.e., around 12 participants in each group) [11]. In contrast, the roughly twice as large sample size in our work provided greater power to detect the differences between the two groups. Lastly, our work measured actual performance via laboratory measures which provide more precise indexes than retrospective questionnaire measures [50] or field tests [49].

The mindfulness training group in our study exhibited longer exhaustion times than the control group, a difference which may be associated with mindfulness-related breathing and posture, which have been reported to decrease task-related worries and task-irrelevant cognitions [11, 50]. Furthermore, Jones and Parker [49] previously demonstrated that pain catastrophizing partially mediates the association between mindfulness and the 800-meter personal best times of runners. Specifically, runners with high levels of mindfulness could have awareness of their pain without judging it, allowing them to accept the experience of it. Mindfulness training also changes physiological responses [44, 51, 52]. For example, Solberg et al. [52] observed that lactate concentration decreased significantly following mindfulness training, and both John et al. [44] and MacDonald and Minahan [51] suggested that mindfulness training decreases salivary cortisol associated with the precompetition or competition period.

### 4.3. Behavioral Performance

Our findings of enhanced Stroop task performance, for both the congruent and incongruent conditions, following mindfulness training are consistent with previous investigations of brief meditation interventions [53]. Given that the two conditions reflect basic information processing and executive functions, respectively [29, 30], the results suggest that mindfulness is positively linked to multiple cognitive functions. In previous studies, experienced meditators or those who received mindfulness meditation training demonstrated better information processing speeds in an attention task [54] or vigilance task [55], suggesting the beneficial effect of mindfulness on basic levels of cognitive function. Our study contributes further to the understanding of the effects of mindfulness because it extends the study of mindfulness effects from the general population to athletes. More importantly, attention is the essential mental ability for peak sport performance, reflecting the potential role of mindfulness in psychological skill training for athletes.

Notably, in typical Stroop effect patterns, longer reaction times and lower accuracy levels are observed in the incongruent condition compared to the congruent condition. That is, in the incongruent condition, individuals must effortfully inhibit more automated processes (i.e., reading a word) to respond in a less automated way (i.e., naming an ink color), such that executive functions must be involved. The linkage between mindfulness and executive functioning indicated in our study is consistent with previous investigations [20, 54], and again, the study further extends the understanding of the executive function benefits of mindfulness training among athletes.

It is possible that mindfulness training helps individuals to focus on the correct targets by repeatedly inhibiting external distractions and improving their ability to ignore any other sources of interference [21, 56], thereby improving their self-regulation in the face of distractions [57], in addition to reducing their negative thoughts (e.g., worries or ruminations) [58]. Indeed, Sanger and Dorjee [59] suggested that mindfulness training for older adolescents enhances their task-relevant inhibitory control of attention and irrelevant interference, in addition to reducing their critical self-judgments. Furthermore, the effects of mindfulness training on inhibitory control can be interpreted from the perspective of brain neuroplasticity. Teper and Inzlicht [20] postulated that the ERN amplitude plays the role of mediator between mindfulness experiences and inhibitory control, as demonstrated in the Stroop task, and that the ERN amplitude reflects the ability to manage, through the anterior cingulate cortex (ACC) activity, the emotions associated with making errors. Experienced meditators have been shown to exhibit increased activation of the medial prefrontal cortex and ACC [60], as well as increased activation of both the medial prefrontal cortex and insula, after mindfulness training compared to nonmeditators [61], with these brain regions having been demonstrated to possibly be involved in the implementation of inhibitory control [62].

The findings of the current study may support previous cross-sectional studies that observed an association between cognitive functions and sport performance [16, 17]. Given that mindfulness training was found to improve Stroop task accuracy and exhaustion time simultaneously, it is possible that mindfulness corresponds with multiple cognitive functions because more mindful athletes prevent or diminish their negative thoughts and experiences. Runners are required to suppress both the physical and psychological symptoms of fatigue when running [15] and to prevent distractions caused by task-irrelevant information in order to achieve excellent running performance [63]. Furthermore, given that stress has been shown to influence executive functions [64] and that mindfulness training appears to reduce stress [65], it is also possible that mindfulness enhances athletes' executive functions and performance via stress reduction. Indeed, meditation practices have been observed to reduce psychological stress responses and improve cognitive functions [66]. It is suggested that future researches be conducted to clarify the relationships among mindfulness, cognitive functions including executive functions, and sport performance by examining the factors of distraction and stress.

### 4.4. N2 of ERP

One of the advances of the current study was its use of the N2 component of ERPs, a sensitive marker of response inhibition that is elicited during conflict tasks [59, 67], to examine the effects of mindfulness on executive functions. While increased N2 amplitudes were observed in both groups in this study, a smaller N2 amplitude in the incongruent condition for the mindfulness group relative to the control group was observed after adjusting for the pre-intervention results as a covariate. A smaller N2 amplitudes have been linked to better neural efficiency within the executive attention networks [57], for example, Zhang, Ouyang, Tang, Chen, and Li [68] suggested that a brief mindfulness intervention resulted in attenuated P1, N2, and LPP amplitudes for positive and negative affective pictures. Furthermore, smaller N2 amplitudes related to the Stroop task following brief meditation training [53] or associated with the AX-Continuous Performance Task following an 8-week mindfulness program [69] have been reported. Nevertheless, previous studies have also observed larger N2 amplitudes associated with mindfulness [56, 59, 70]. It should be noted, however, that while Moore et al. [56] found that mindfulness meditation led to increased N2 amplitudes during the Stroop task, these larger N2 amplitudes were not accompanied by improvements in behavioral performance. Similarly, Sanger and Dorjee [59] observed more negative N2 amplitudes after mindfulness training in adolescents, but they did not observe any changes in attention task performance. Therefore, our finding of smaller N2 amplitudes for the incongruent condition accompanied by better behavioral performance after mindfulness training in the present study may be associated with reduced activity related to conflict monitoring and more efficient cognitive control mechanisms, which may in turn suggest that mindfulness training enables more rapid and accurate processing of stimuli [71, 72].

### 4.5. Limitations and Future Directions

The results of this study should be interpreted with caution, as the study had several limitations that must be taken into account. First, a quasiexperimental design was employed, that is, the assignment of the participants to the mindfulness group or the control group was not fully random, which would limit the further interpretation of causality. Relatedly, larger sample sizes of athletes from various sports are encouraged in future researches in order to determine the generalizability of our findings. Second, the approach used for the control group in this study is a matter of some controversy. Specifically, we utilized a waitlist-control group, which is beneficial in terms of providing initial evidence; however, the use of active control groups, including those receiving relaxation training or other typical psychological skills training, could be problematic because of possible placebo effects [73]. Finally, mindfulness in our study was measured using self-report inventories, the responses to which could be subject to social desirability and subjective bias effects. Relatedly, it has been suggested that studies should adopt standardized objective measures of mindfulness, such as the breath-counting task, in order to objectively represent mindfulness [74, 75].

## 5. Conclusion

The present study provides initial evidence regarding the relationships among a mindfulness intervention, sport performance, and executive functions in athletes from both behavioral and electrophysiological perspectives. This investigation demonstrated that a five-week mindfulness training program with two 30 min practice sessions per week positively affected the participating athletes' endurance performance and cognitive functions, including executive functions, in addition to increasing the efficiency of conflict resolution in neural processes.

## Figures and Tables

**Table 1:** Baseline characteristics of the mindfulness and control groups (*M* ± SD).

Variables	Mindfulness group (*n* = 23)	Control group (*n* = 23)	*t*	*p*
Gender (M/F)	16/7	18/5	—	—
Age (years)	19.83 ± 1.44	20.04 ± 1.55	-.493	.624
Height (cm)	166.43 ± 6.34	174.60 ± 8.23	-3.767	.001
Body mass (kg)	61.07 ± 7.94	64.87 ± 9.09	-1.512	.138
BMI (kg/m^2^)	21.98 ± 2.04	21.18 ± 1.49	1.516	.137
Education (years)	14.35 ± 1.27	14.17 ± 1.07	.503	.618
Sport experience (years)	8.91 ± 3.65	10.02 ± 2.89	-1.142	.260
Digit span test				
Forward	14.65 ± 1.58	14.22 ± 1.98	.823	.415
Backward	8.43 ± 2.97	7.83 ± 2.37	.769	.446

Note. BMI: body mass index; M: male; F: female.

**Table 2:** The performance of mindfulness, sport, behavioral, and ERP indices for the two groups before and after the mindfulness training program (*M* ± SD).

Variables	Mindfulness group (*n* = 23)		Control group (*n* = 23)	
	Pretest	Posttest	Pretest	Posttest
Mindfulness	4.52 ± 0.65	4.71 ± 0.52	4.45 ± 0.59	4.38 ± 0.68
GXT				
VO_2max_	51.64 ± 5.08	52.60 ± 4.67	56.53 ± 5.40	56.49 ± 6.16
Exhaust. (s)	584.70 ± 97.81	641.22 ± 90.46	682.48 ± 101.19	674.35 ± 94.31
Reaction time (ms)				
Cong.	598.11 ± 85.50	580.22 ± 71.68	543.59 ± 76.25	530.85 ± 70.18
Incong.	664.52 ± 120.96	630.33 ± 95.09	593.37 ± 105.30	557.37 ± 81.82
Accuracy (%)				
Cong.	93.96 ± 4.10	95.70 ± 2.36	93.56 ± 6.55	92.15 ± 6.74
Incong.	87.35 ± 10.63	92.61 ± 5.40	89.21 ± 8.39	88.57 ± 8.99
N2 amplitude (*μ*V)				
Cong.	−3.28 ± 5.43	−5.40 ± 5.39	0.37 ± 3.75	−3.47 ± 3.57
Incong.	−3.32 ± 5.44	−5.04 ± 5.34	0.78 ± 3.51	−3.14 ± 3.39

Note. GXT: graded exercise test; VO_2max_ (mL/kg/min); Exhaust.: exhaustion time; Cong.: congruent condition; Incong.: incongruent condition.

## Data Availability

The authors will be glad to share the data by requested.

## Appendix

### Summary Outline of the Five-Week Mindfulness Protocol

(I) Session 1
  (A) Introduction of mindfulness
    1. Review of key mental factors in athletic competitions
    2. Introduction of mindfulness practice
    3. Introduction and psychoeducation regarding the mindfulness program
  (B) Introduction of mindful breathing
    1. Explanation of a mindful breathing exercise
    2. Group discussion
  (C) Discussion of homework
(II) Session 2
  (A) Warm-up: 5 min mindful breathing exercise
  (B) Introduction of the components of mindfulness
    1. Awareness
  (C) Introduction of mindfulness meditation
    1. Explanation of a mindfulness meditation exercise
    2. Group discussion
  (D) Discussion of homework
(III) Session 3
  (A) Warm-up: 5 min mindful breathing exercise
  (B) Introduction of the components of mindfulness
    1. Nonjudgement
  (C) Introduction of body scanning
    1. Explanation of a body scanning exercise
    2. Group discussion
  (D) Discussion of homework
(IV) Session 4
  (A) Warm-up: 5 min mindful breathing exercise
  (B) Introduction of the components of mindfulness
    1. Acceptance
  (C) Introduction of mindful yoga
    1. Explanation of a mindful yoga exercise
    2. Group discussion
  (D) Discussion of homework
(V) Session 5
  (A) Warm-up: 5 min mindful breathing exercise
  (B) Introduction of the components of mindfulness
    1. Curiosity
  (C) Introduction of mindful walking
    1. Explanation of a mindful walking exercise
    2. Group discussion
  (D) Discussion of homework
(VI) Session 6
  (A) Warm-up: 5 min mindful breathing exercise
  (B) How is mindfulness applied to sport?
    1. Mindful breathing before a competition
    2. Being mindful of perceptions of stress
  (C) Performance of a mindfulness meditation exercise
  (D) Group discussion and homework
(VII) Session 7
  (A) Warm-up: 5 min mindful breathing exercise
  (B) How is mindfulness applied to sport
    1. Self-awareness before a competition
  (C) Performance of a body scanning exercise
  (D) Group discussion and homework
(VIII) Session 8
  (A) Warm-up: 5 min mindful breathing exercise
  (B) How is mindfulness applied to sport
    1. Focusing on physical perceptions before a competition
  (C) Performance of a mindful yoga exercise
  (D) Group discussion and homework
(IX) Session 9
  (A) Warm-up: 5 min mindful breathing exercise
  (B) How is mindfulness applied to sport
    1. Nonjudgement of negative thoughts before a competition
  (C) Performance of a mindful walking exercise
  (D) Group discussion and homework
(X) Session 10
  (A) Warm-up: 5-min mindful breathing exercise
  (B) How is mindfulness applied to sport
    1. Accepting negative thoughts before a competition
  (C) Performance of a mindfulness meditation exercise
  (D) Summary
    1. What are the benefits mindfulness for you
    2. Discussion of strategies for continued practice

## References

[1] Crane R. S., Brewer J., Feldman C. (2017). What defines mindfulness-based programs? The warp and the weft. *Psychological Medicine*.

[2] Ludwig D. S., Kabat-Zinn J. (2008). Mindfulness in medicine. *JAMA*.

[3] Kabat-Zinn J. (2006). Mindfulness-based interventions in context: past, present, and future. *Clinical Psychology: Science and Practice*.

[4] Creswell J. D. (2017). Mindfulness interventions. *Annual Review of Psychology*.

[5] Cillessen L., Johannsen M., Speckens A. E. M., Zachariae R. (2019). Mindfulness-based interventions for psychological and physical health outcomes in cancer patients and survivors: a systematic review and meta-analysis of randomized controlled trials. *Psychooncology*.

[6] Vignaud P., Donde C., Sadki T., Poulet E., Brunelin J. (2018). Neural effects of mindfulness-based interventions on patients with major depressive disorder: a systematic review. *Neuroscience and Biobehavioral Reviews*.

[7] Buhlmayer L., Birrer D., Rothlin P., Faude O., Donath L. (2017). Effects of mindfulness practice on performance-relevant parameters and performance outcomes in sports: a meta-analytical review. *Sports Medicine*.

[8] Pineau T. R., Glass C. R., Kaufman K. A. (2014). Mindfulness in sport performance. *The Wiley Blackwell handbook of mindfulness*.

[9] Cathcart S., McGregor M., Groundwater E. (2014). Mindfulness and flow in elite athletes. *Journal of Clinical Sport Psychology*.

[10] Zhang C. Q., Chung P. K., Si G. (2017). Assessing acceptance in mindfulness with direct-worded items: the development and initial validation of the Athlete Mindfulness Questionnaire. *Journal of Sport and Health Science*.

[11] De Petrillo L. A., Kaufman K. A., Glass C. R., Arnkoff D. B. (2009). Mindfulness for long-distance runners: an open trial using Mindful Sport Performance Enhancement (MSPE). *Journal of Clinical Sport Psychology*.

[12] Diamond A. (2013). Executive functions. *Annual Review of Psychology*.

[13] Friedman N. P., Miyake A. (2017). Unity and diversity of executive functions: individual differences as a window on cognitive structure. *Cortex*.

[14] Ambrosini E., Arbula S., Rossato C., Pacella V., Vallesi A. (2019). Neuro-cognitive architecture of executive functions: a latent variable analysis. *Cortex*.

[15] Jacobson J., Matthaeus L. (2014). Athletics and executive functioning: how athletic participation and sport type correlate with cognitive performance. *Psychology of Sport and Exercise*.

[16] Verburgh L., Scherder E. J. A., van Lange P. A. M., Oosterlaan J. (2014). Executive functioning in highly talented soccer players. *PLoS One*.

[17] Huijgen B. C. H., Leemhuis S., Kok N. M. (2015). Cognitive functions in elite and sub-elite youth soccer players aged 13 to 17 years. *PLoS One*.

[18] Gallant S. N. (2016). Mindfulness meditation practice and executive functioning: breaking down the benefit. *Consciousness and Cognition*.

[19] Moynihan J. A., Chapman B. P., Klorman R. (2013). Mindfulness-based stress reduction for older adults: effects on executive function, frontal alpha asymmetry and immune function. *Neuropsychobiology*.

[20] Teper R., Inzlicht M. (2013). Meditation, mindfulness and executive control: the importance of emotional acceptance and brain-based performance monitoring. *Social Cognitive and Affective Neuroscience*.

[21] Allen M., Dietz M., Blair K. S. (2012). Cognitive-affective neural plasticity following active-controlled mindfulness intervention. *The Journal of Neuroscience*.

[22] Quaglia J. T., Zeidan F., Grossenbacher P. G. (2019). Brief mindfulness training enhances cognitive control in socioemotional contexts: behavioral and neural evidence. *PLoS One*.

[23] Wechsler D. (1997). *Wechsler Adult Intelligence Scale*.

[24] Beltz N. M., Gibson A. L., Janot J. M., Kravitz L., Mermier C. M., Dalleck L. C. (2016). Graded exercise testing protocols for the determination of VO2max: historical perspectives, progress, and future considerations. *Journal of Sports Medicin*.

[25] Borg G. A. V. (1982). Psychophysical bases of perceived exertion. *Medicine and Science in Sports and Exercise*.

[26] Ho C. F., Shih C. Y., Chan K. H., Wang T. Y. (2012). Effect of uphill high-intensity interval training on aerobic capacity and power of lower extremity in basketball players. *Sports & Exercise Research*.

[27] Yang M. T., Lee M. M., Hsu S. C., Chan K. H. (2017). Effects of high-intensity interval training on canoeing performance. *European Journal of Sport Science*.

[28] Stroop J. R. (1935). Studies of interference in serial verbal reactions. *Journal of Experimental Psychology*.

[29] Etnier J. L., Chang Y. K. (2009). The effect of physical activity on executive function: a brief commentary on definitions, measurement issues, and the current state of the literature. *Journal of Sport & Exercise Psychology*.

[30] Miyake A., Friedman N. P., Emerson M. J., Witzki A. H., Howerter A., Wager T. D. (2000). The unity and diversity of executive functions and their contributions to complex “frontal lobe” tasks: a latent variable analysis. *Cognitive Psychology*.

[31] Chang J. H., Lin Y. C., Huang C. L. (2011). Psychometric properties of the Chinese Translation of Mindful Attention Awareness Scale (CMAAS). *Psychol Test*.

[32] Brown K. W., Ryan R. M. (2003). The benefits of being present: mindfulness and its role in psychological well-being. *Journal of Personality and Social Psychology*.

[33] Kabat-Zinn J. (1982). An outpatient program in behavioral medicine for chronic pain patients based on the practice of mindfulness meditation: theoretical considerations and preliminary results. *General Hospital Psychiatry*.

[34] Kaufman K. A., Glass C. R. (2006). *Mindful Sport Performance Enhancement: a treatment manual for archers and golfers*.

[35] Bland J. M., Altman D. G. (2011). Comparisons against baseline within randomised groups are often used and can be highly misleading. *Trials*.

[36] Clifton L., Clifton D. A. (2019). The correlation between baseline score and post-intervention score, and its implications for statistical analysis. *Trials*.

[37] de Boer M. R., Waterlander W. E., Kuijper L. D., Steenhuis I. H., Twisk J. W. (2015). Testing for baseline differences in randomized controlled trials: an unhealthy research behavior that is hard to eradicate. *International Journal of Behavioral Nutrition and Physical Activity*.

[38] Xunlin N. G., Lau Y., Klainin-Yobas P. (2020). The effectiveness of mindfulness-based interventions among cancer patients and survivors: a systematic review and meta-analysis. *Support Care Cancer*.

[39] Zhang Q., Zhao H., Zheng Y. (2019). Effectiveness of mindfulness-based stress reduction (MBSR) on symptom variables and health-related quality of life in breast cancer patients-a systematic review and meta-analysis. *Support Care Cancer*.

[40] Jansen J. E., Gleeson J., Bendall S., Rice S., Alvarez-Jimenez M. (2020). Acceptance- and mindfulness-based interventions for persons with psychosis: a systematic review and meta-analysis. *Schizophrenia Research*.

[41] Haugmark T., Hagen K. B., Smedslund G., Zangi H. A. (2019). Mindfulness- and acceptance-based interventions for patients with fibromyalgia - a systematic review and meta-analyses. *PLoS One*.

[42] Aherne C., Moran A. P., Lonsdale C. (2011). The effect of mindfulness training on athletes’ flow: an initial investigation. *Sport Psychologist*.

[43] Mehrsafar A. H., Strahler J., Gazerani P. (2019). The effects of mindfulness training on competition-induced anxiety and salivary stress markers in elite Wushu athletes: a pilot study. *Physiology & Behavior*.

[44] John S., Verma S. K., Khanna G. L. (2011). The effect of mindfulness meditation on HPA-Axis in pre-competition stress in sports performance of elite shooters. *National Journal of Integrated Research in Medicine*.

[45] Chen J. H., Tsai P. H., Lin Y. C., Chen C. K., Chen C. Y. (2019). Mindfulness training enhances flow state and mental health among baseball players in Taiwan. *Psychology Research and Behavior Management*.

[46] Kaufman K. A., Glass C. R., Arnkoff D. B. (2009). Evaluation of mindful sport performance enhancement (MSPE): a new approach to promote flow in athletes. *Journal of Clinical Sport Psychology*.

[47] Gooding A., Gardner F. L. (2009). An investigation of the relationship between mindfulness, preshot routine, and basketball free throw percentage. *Journal of Clinical Sport Psychology*.

[48] Zhang C.-Q., Si G., Duan Y., Lyu Y., Keatley D. A., Chan D. K. C. (2016). The effects of mindfulness training on beginners' skill acquisition in dart throwing: a randomized controlled trial. *Psychology of Sport and Exercise*.

[49] Jones M. I., Parker J. K. (2015). A conditional process model of the effect of mindfulness on 800-m personal best times through pain catastrophising. *Journal of Sports Sciences*.

[50] Thompson R. W., Kaufman K. A., De Petrillo L. A., Glass C. R., Arnkoff D. B. (2011). One year follow-up of Mindful Sport Performance Enhancement (MSPE) with archers, golfers, and runners. *Journal of Clinical Sport Psychology*.

[51] MacDonald L. A., Minahan C. L. (2017). Mindfulness training attenuates the increase in salivary cortisol concentration associated with competition in highly trained wheelchair-basketball players. *Journal of Sports Sciences*.

[52] Solberg E. E., Ingjer F., Holen A., Sundgot-Borgen J., Nilsson S., Holme I. (2000). Stress reactivity to and recovery from a standardised exercise bout: a study of 31 runners practising relaxation techniques. *British Journal of Sports Medicine*.

[53] Fan Y., Tang Y. Y., Tang R., Posner M. I. (2015). Time course of conflict processing modulated by brief meditation training. *Frontiers in Psychology*.

[54] Moore A., Malinowski P. (2009). Meditation, mindfulness and cognitive flexibility. *Consciousness and Cognition*.

[55] Ching H.-H., Koo M., Tsai T.-H., Chen C.-Y. (2015). Effects of a mindfulness meditation course on learning and cognitive performance among university students in Taiwan. *Evidence-based Complementary and Alternative Medicine*.

[56] Moore A., Gruber T., Derose J., Malinowski P. (2012). Regular, brief mindfulness meditation practice improves electrophysiological markers of attentional control. *Frontiers in Human Neuroscience*.

[57] Kaunhoven R. J., Dorjee D. (2017). How does mindfulness modulate self-regulation in pre-adolescent children? An integrative neurocognitive review. *Neuroscience & Biobehavioral Reviews*.

[58] Chesin M. S., Benjamin-Phillips C. A., Keilp J., Fertuck E. A., Brodsky B. S., Stanley B. (2016). Improvements in executive attention, rumination, cognitive reactivity, and mindfulness among high-suicide risk patients participating in adjunct mindfulness-based cognitive therapy: preliminary findings. *Journal of Alternative and Complementary Medicine*.

[59] Sanger K. L., Dorjee D. (2016). Mindfulness training with adolescents enhances metacognition and the inhibition of irrelevant stimuli: evidence from event-related brain potentials. *Trends in Neuroscience and Education*.

[60] Hölzel B. K., Ott U., Hempel H. (2007). Differential engagement of anterior cingulate and adjacent medial frontal cortex in adept meditators and non-meditators. *Neuroscience Letters*.

[61] Haase L., May A. C., Falahpour M. (2015). A pilot study investigating changes in neural processing after mindfulness training in elite athletes. *Frontiers in Behavioral Neuroscience*.

[62] Grandjean J., D’Ostilio K., Phillips C. (2012). Modulation of brain activity during a Stroop inhibitory task by the kind of cognitive control required. *PLoS One*.

[63] Cona G., Cavazzana A., Paoli A., Marcolin G., Grainer A., Bisiacchi P. S. (2015). It’s a matter of mind! Cognitive functioning predicts the athletic performance in ultra-marathon runners. *PLoS One*.

[64] Henderson R. K., Snyder H. R., Gupta T., Banich M. T. (2012). When does stress help or harm? The effects of stress controllability and subjective stress response on Stroop performance. *Frontiers in Psychology*.

[65] Vibe M., Bjørndal A., Fattah S., Dyrdal G. M., Halland E., Tanner-Smith E. E. (2017). Mindfulness-based stress reduction (MBSR) for improving health, quality of life and social functioning in adults: a systematic review and meta-analysis. *Campbell Systematic Reviews*.

[66] Singh Y., Sharma R., Talwar A. (2012). Immediate and long-term effects of meditation on acute stress reactivity, cognitive functions, and intelligence. *Alternative Therapies in Health and Medicine*.

[67] Ladouceur C. D., Dahl R. E., Carter C. S. (2007). Development of action monitoring through adolescence into adulthood: ERP and source localization. *Developmental Science*.

[68] Zhang W., Ouyang Y., Tang F., Chen J., Li H. (2019). Breath-focused mindfulness alters early and late components during emotion regulation. *Brain and Cognition*.

[69] Incagli F., Tarantino V., Crescentini C., Vallesi A. (2020). The effects of 8-week mindfulness-based stress reduction program on cognitive control: an EEG study. *Mindfulness*.

[70] Malinowski P., Moore A. W., Mead B. R., Gruber T. (2017). Mindful aging: the effects of regular brief mindfulness practice on electrophysiological markers of cognitive and affective processing in older adults. *Mindfulness*.

[71] Finkenzeller T., Doppelmayr M., Würth S., Amesberger G. (2018). Impact of maximal physical exertion on interference control and electrocortical activity in well-trained persons. *European Journal of Applied Physiology*.

[72] Isbel B. D., Lagopoulos J., Hermens D. F., Summers M. J. (2019). Mental training affects electrophysiological markers of attention resource allocation in healthy older adults. *Neuroscience Letters*.

[73] Enck P., Zipfel S. (2019). Placebo effects in psychotherapy: a framework. *Frontiers in Psychiatry*.

[74] Levinson D. B., Stoll E. L., Kindy S. D., Merry H. L., Davidson R. J. (2014). A mind you can count on: validating breath counting as a behavioral measure of mindfulness. *Frontiers in Psychology*.

[75] Wong K. F., Massar S. A. A., Chee M. W. L., Lim J. (2018). Towards an objective measure of mindfulness: replicating and extending the features of the breath-counting task. *Mindfulness*.

